# Unvaporized e-liquid toxicity elevates CD44-dependent hyaluronan catabolic gene expression and triggers inflammation in human vocal fold fibroblasts

**DOI:** 10.1016/j.toxrep.2025.102152

**Published:** 2025-10-28

**Authors:** Kaustuv Basu, James Li, Luc Mongeau

**Affiliations:** Department of Mechanical Engineering, McGill University, Montreal, QC, Canada

**Keywords:** Voice, E-cigarettes, Toxicity, Collagen, HYAL2, CD44, Hyaluronic acid, Inflammation

## Abstract

Electronic (e)-cigarette and e-liquid exposure have been linked to vocal fold inflammation and dysphonia, yet no targeted non-surgical therapies currently exist. Hyaluronan, a key extracellular matrix component essential for vocal fold structure, repair, and function, is known to be dysregulated in inflammatory conditions; however, its metabolic gene response to e-liquid exposure in human vocal fold fibroblasts (hVFFs) remains uncharacterized. Hence, it is critical to understand hyaluronan metabolic gene expression under e-liquid toxicity to develop novel drug discovery strategies for vocal fold inflammation. To avoid confounding effects from thermal degradation and aerosol variability in conventional vapor models, hVFFs were exposed to nicotine-containing unvaporized e-liquid (0.125–1 mg/mL) for 24 h, revealing concentration-dependent changes in cell morphology and viability (p < 0.05). The lethal concentration 50 (LC₅₀) was determined to be 0.437 mg/mL and used for short-term (24 h) and extended (72–96 h) exposures. Extended exposure induced intracellular reactive oxygen species (ROS), inflammation, suppressed collagenolysis, and increased the collagen 1 A: collagen 3 A ratio, suggesting fibrotic remodeling. Short-term exposure downregulated hyaluronan synthases (HAS1, HAS2, HAS3) and catabolic genes (HYAL2, CD44), reducing extracellular hyaluronan levels. In contrast, extended exposure repressed HAS1 and HAS2 while upregulating HAS3, CD44, and HYAL2, indicating enhanced hyaluronan degradation and accumulation of proinflammatory low molecular weight hyaluronan. CD44 silencing reduced *IL-8* mRNA expression, confirming its role in hVFF inflammation. These findings provide the first mechanistic insight into unvaporized e-liquid-induced dysregulation of hyaluronan metabolism in hVFFs, offering a foundation for biomarker identification and therapeutic development targeting e-cigarette-associated vocal fold inflammation.

## Highlights

• No targeted therapy for vocal fold inflammation from e-liquid toxicity.
• Hyaluronan (HA) is vital for vocal fold structure, repair, and proper function.
• No data on HA metabolic gene expression in vocal fold fibroblast (VFF) in e-liquid.
• First study on unvaporized e-liquid’s effect on HA metabolic genes in human VFFs.
• E-liquid boosts CD44-linked low MW HA levels, triggering VFF inflammation.

## Graphical Abstract

**Figure:**
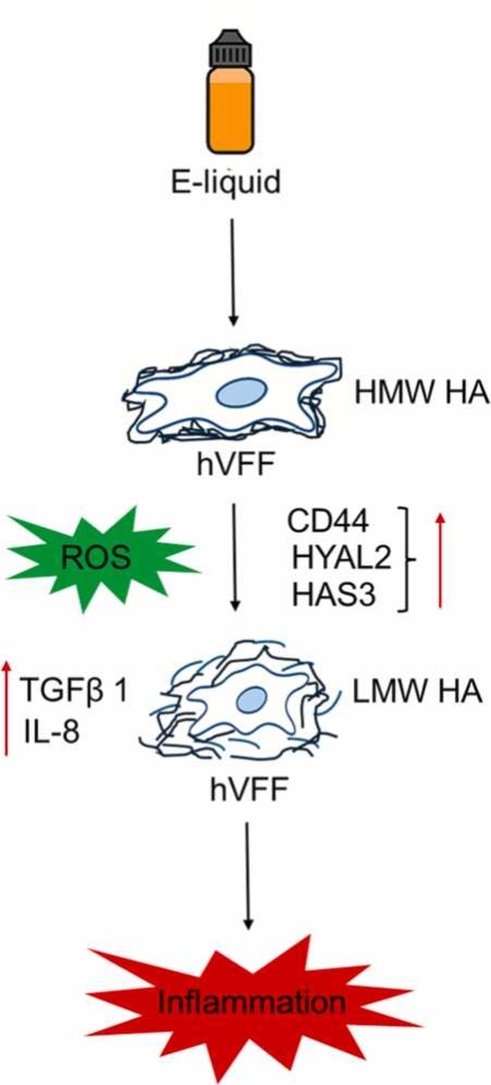

## 1 Introduction

The rapid rise of electronic nicotine delivery systems (ENDS), or electronic (e)-cigarettes induce significant pathological changes in the respiratory tract, particularly the vocal folds, resulting in hoarseness, vocal fatigue, and the development of nodules, polyps, Reinke’s edema (vocal fold edema) and increase cancer risk, underscoring the urgent need for mechanistic research [1], [2], [3], [4], [5], [6], [7]. E-cigarettes contain e-liquid composed of nicotine, flavoring agents, and carrier solvents such as propylene glycol (PG) and vegetable glycerin (VG). Upon vaporization, these constituents undergo thermal degradation, producing harmful byproducts including formaldehyde, acetaldehyde, and acrolein, many of which are known carcinogens [8], [9], [10]. Aerosolized e-liquids contain significantly more toxic compounds than their unheated counterparts, with the number of chemical constituents increasing post-vaporization due to device-specific heating conditions [11]. Consequently, unvaporized e-liquids are less chemically reactive and offer a more stable substrate for controlled toxicological screening. Direct use of e-liquid formulations enables precise control over constituent concentrations, facilitating reproducible dosing and targeted assessment of individual ingredients such as nicotine, flavoring agents, and PG/VG [12]. Moreover, unvaporized e-liquid avoids the variability introduced by aerosol generation.

Human vocal fold fibroblasts (hVFF) constitute the prevalent cell type in the lamina propria, the subepithelial layer of the human vocal fold mucosa. They are essential mediators of inflammation and play a critical role in maintaining the extracellular matrix (ECM) of the vocal folds [13], [14], [15], [16], [17]. hVFFs are particularly vulnerable during vocal fold scarring from e-cigarettes [18]. Among ECM components, hyaluronan, a viscoelastic glycosaminoglycan, is abundantly present in the lamina propria and regulates phonation, hydration, and tissue integrity [19], [20]. Hyaluronan is synthesized by three isoforms of hyaluronan synthase (HAS), encoded by *HAS1, HAS2*, and *HAS3*. Under physiological conditions, hVFFs predominantly express HAS2, facilitating the production of HMW HA, which supports tissue hydration, reduces friction during vocal fold vibration, and suppresses inflammatory signaling [17], [19]. However, in pathological states such as scarring or exposure to environmental toxins, VFFs may shift toward increased HAS3 expression, leading to elevated levels of LMW HA, which promotes fibroblast activation, cytokine release, and ECM stiffening [21], [22]. This shift in hyaluronan metabolism is often accompanied by upregulation of CD44 and hyaluronidases, further amplifying inflammatory cascades and tissue remodeling [23], [24], [25], [26]. The transcriptional regulation of HAS enzymes is influenced by factors such as p53, p63, and Transforming Growth Factor Beta 1 (TGF-β1), which modulate hyaluronan synthesis in response to cellular stress and injury [27], [28]. Understanding the molecular mechanisms governing hyaluronan synthesis and degradation in hVFFs is therefore critical for elucidating the pathophysiology of vocal fold disorders and developing targeted therapeutic interventions.

Hyaluronan exerts its biological effects through binding to its principal receptor, CD44, which exists in two major isoforms: standard (CD44s) and variant (CD44v) [29], [30], [31]. These isoforms activate distinct signaling pathways involved in cell proliferation, migration, and inflammation [24], [25]. Hyaluronan degradation occurs via two primary mechanisms: (a) through hyaluronan catabolic gene activity in a CD44-dependent pathway involving the hyaluronidases (HYAL): HYAL1 and HYAL2, and a CD44-independent pathway; and (b) through oxidative cleavage mediated by reactive oxygen species (ROS) [31], [32], [34]. In the CD44-dependent route, HYAL2 cleaves extracellular hyaluronan into intermediate fragments, which are internalized and further degraded by HYAL1 within lysosomes [26]. While the effects of conventional cigarette smoke on hyaluronan metabolism in hVFFs have been documented, there is currently no published report on how e-cigarette or e-liquid exposure affects the expression of genes regulating hyaluronan synthesis and degradation in hVFFs. The present study aims to investigate the toxic effects of unvaporized e-liquids on the mRNA expression of hyaluronan metabolic genes in immortalized hVFF cells *in vitro*, providing mechanistic insight into ECM dysregulation and vocal fold pathology associated with vaping.

## 2 Materials and Methods

### 2.1 Cell culture

Human immortalized vocal fold fibroblast (hVFF) cells were generously provided by Prof. Susan Thibeault from the University of Wisconsin–Madison, USA, as previously described by Ling et al. [35]. Cells were cultured in Dulbecco’s Modified Eagle Medium (DMEM; Thermo Fisher Scientific, Cat# 21068028) containing 4.5 g/L glucose, supplemented with 10 % fetal bovine serum (FBS; Sigma-Aldrich, Cat# F0926) and 1 % penicillin-streptomycin to support cell growth and prevent microbial contamination. Cultures were maintained in a humidified incubator at 37 °C with 5 % CO₂, and the medium was refreshed every 2–3 days to ensure optimal nutrient availability and cell viability. For subculturing or experimental harvesting, cells were gently rinsed with phosphate-buffered saline (PBS; pH 7.2–7.4) to remove residual medium and detached using TrypLE™ Express Enzyme (1X; Gibco, Cat# 12563011), a recombinant, animal origin-free alternative to trypsin that preserves cell surface proteins (e.g., CD44).

### 2.2 Source and pre-analysis treatment of e-liquid

Green apple-flavored e-liquid (Mucho brand; 100 mL bottle; nicotine concentration: 3 mg/mL; composition: vegetable glycerin (VG) 70 %, propylene glycol (PG) 30 %, natural and artificial flavorings, and nicotine) was procured from a local retail outlet in Montreal, Canada in a sealed bottle, labelled by the manufacturer. E-liquid of this flavor and brand was selected based on its inclusion in the fruit flavor category, which ranks among the top five most popular e-liquid flavor types sold in Canada [36]. To prepare the treatment solution, the e-liquid was mixed with serum-free Dulbecco’s Modified Eagle Medium (DMEM) and incubated overnight at room temperature to ensure homogenous dispersion of its constituents. The resulting stock solution was subsequently diluted in complete growth medium to achieve the desired working concentrations of nicotine and other e-liquid components. hVFFs were exposed to the diluted unvaporized e-liquid (0.125–1 mg/mL) for 24 h, yielding the lethal concentration 50 (LC₅₀) of 0.437 mg/mL under standard culture conditions. Following treatment, cellular morphology was examined using an inverted light microscope in brightfield mode at 20 × magnification. Morphological changes were documented to assess potential cytotoxic or phenotypic alterations induced by e-liquid exposure.

### 2.3 Cell viability assay

Cell viability was evaluated using the WST-1 assay, a colorimetric method based on the enzymatic cleavage of the tetrazolium salt WST-1 to soluble formazan by mitochondrial dehydrogenases in metabolically active cells. hVFF cells were seeded into 96-well plates, treated with unvaporized e-liquid (0.125–1 mg/mL) for 24 h followed by addition of WST-1 proliferation reagent (CELLPRO-RO; Sigma-Aldrich, Cat# 05015944001) at a volume of 10 µL per well. This reagent contains both the WST-1 substrate and an electron coupling reagent that facilitates the reduction process. Following reagent addition, plates were incubated at 37 °C in a humidified atmosphere containing 5 % CO₂ for 2–4 h in the dark to prevent light-induced degradation of the dye, followed by quick mixing on an orbital shaker for 1 min. The amount of formazan dye generated, which correlates directly with the number of viable cells, was quantified by measuring absorbance at 440 nm using a microplate (ELISA) reader. A reference wavelength above 600 nm was used to correct for non-specific background absorbance. All measurements were performed in triplicate, and results are expressed as mean ± standard deviation (SD) from three independent experimental replicates.

### 2.4 Reactive oxygen species (ROS) assay

Intracellular reactive oxygen species (ROS) generation in human vocal fold fibroblast (hVFF) cells was assessed using the fluorescent probe 2′,7-dichlorofluorescin diacetate (DCFH-DA; Sigma-Aldrich, Cat# D6665), following previously described protocols. Cells were treated with or without e-liquid (0.437 mg/mL for 72 h) and hydrogen peroxide (H₂O₂, 5 µM) as a positive control to induce oxidative stress. Following treatment, cells were incubated with DCFH-DA at a final concentration of 5 µg/mL for 10 min at 37 °C in the dark to prevent photobleaching. DCFH-DA is a non-fluorescent compound that diffuses into cells and is hydrolyzed by intracellular esterases to DCFH, which is subsequently oxidized by ROS to yield the highly fluorescent compound 2′,7-dichlorofluorescein (DCF). After incubation, the probe was removed, and cells were washed twice with ice-cold phosphate-buffered saline (PBS; pH 7.4; ThermoFisher Scientific, Cat# 10010023) to eliminate excess dye and minimize background fluorescence. Fluorescence intensity (FI) was measured using a microplate reader at an excitation wavelength of 488 nm and an emission wavelength of 530 nm. To account for variations in cell density and metabolic activity, the cells were scraped and following exposure to heat and cold temperatures alternatively, total protein concentrations were quantified using NanoDrop™ spectrophotometer. FI values were normalized to total protein concentration (mg/mL). All measurements were performed in triplicate, and data are presented as mean ± standard deviation (SD) from three replicates.

### 2.5 Quantitative real-time polymerase chain reaction (qRT-PCR)

Total RNA was extracted from cultured human vocal fold fibroblasts (hVFF) using the TRIzol™ reagent (Thermo Fisher Scientific, USA), following the manufacturer’s protocol and previously described procedures [37]. The purity and concentration of RNA were assessed spectrophotometrically, and only samples with an A260/A280 ratio between 1.8 and 2.0 were used for downstream analysis. Complementary DNA (cDNA) was synthesized from 1 µg of total RNA using the iScript™ Reverse Transcription Supermix (Bio-Rad, Cat# 1708890), which includes both oligo(dT) and random primers to ensure comprehensive transcript coverage. Quantitative real-time PCR (qRT-PCR) was performed in triplicate using the SYBR® Green Fast Master Mix (Bio-Rad, Cat# 1725150) on a CFX96 Real-Time PCR Detection System (Bio-Rad Laboratories, USA). The thermal cycling conditions were as follows: initial denaturation at 95 °C for 2 min, followed by 40 amplification cycles of denaturation at 95 °C for 10 s and annealing/extension at 60 °C for 30 s. Gene-specific primers were synthesized by Thermo Fisher Scientific (USA), and sequences are listed in Table 1. Relative gene expression levels were calculated using the ΔΔCt method, with hypoxanthine phosphoribosyltransferase 1 (*HPRT1*) and TATA-box binding protein (*TBP*) serving as the endogenous reference gene for normalization. Data are presented as mean ± standard deviation (SD) from three replicates.

**Table 1:** The list of primers for qRT-PCR analysis.

**Gene**	**Accession No.**	**Forward primer**	**Reverse primer**
*HAS1*	NM_001297436.2	GAGCCTCTTCGCGTACCTG	CCTCCTGGTAGGCGGAGAT
*HAS2*	NM_005328.3	CTCTTTTGGACTGTATGGTGCC	AGGGTAGGTTAGCCTTTTCACA
*HAS3*	NM_001199280.2	CAGCCTATGTGACGGGCTAC	CCTCCTGGTATGCGGCAAT
*CD44*	NM_000610.4	CTGCCGCTTTGCAGGTGTA	CATTGTGGGCAAGGTGCTATT
*HYAL2*	NM_003773.5	GGCCCCACCGTTACATTGG	ATTCTGGTTCACAAAACCCTCAT
*TGFB1*	NM_000660.7	GGCCAGATCCTGTCCAAGC	GTGGGTTTCCACCATTAGCAC
*IL−8*	NM_000584.4	AAGAGAGCTCTGTCTGGACC	GATATTCTCTTGGCCCTTGG
*COL1A*	NM_000088.4	GAGGGCCAAGACGAAGACATC	CAGATCACGTCATCGCACAAC
*COL3A*	NM_000090.4	GGAGCTGGCTACTTCTCGC	GGGAACATCCTCCTTCAACAG
*HPRT1*	NM_000194.3	CCTGGCGTCGTGATTAGTGAT	AGACGTTCAGTCCTGTCCATAA
*TBP*	NM_003194.5	TGGCGTGTGAAGATAACCCAA	TCTTGGCAAACCAGAAACCCT

### 2.6 Collagenase assay

Collagenase activity was quantified using a commercially available collagenase assay kit (Abcam, Cat# ab196999), following the manufacturer’s protocol. This colorimetric assay is based on the cleavage of a synthetic peptide substrate, N-[3-(2-furyl)acryloyl]-Leu-Gly-Pro-Ala (FALGPA), which structurally mimics native collagen and serves as a specific target for collagenase enzymes. A collagenase reaction mixture was freshly prepared by combining the substrate with assay buffer, and aliquots were dispensed into individual wells of a 96-well microplate. Samples containing collagenase were added to the wells and gently mixed to ensure homogeneity. The enzymatic reaction was initiated immediately, and absorbance was recorded at 345 nm using a microplate reader operating in kinetic mode. Measurements were taken continuously for 5–15 min at 37 °C, with the plate protected from direct light to prevent photodegradation of reagents. The rate of decrease in absorbance over time was directly proportional to collagenase activity. All reactions were performed in triplicate, and data are expressed as mean ± standard deviation (SD) from three replicates.

### 2.7 Total extracellular hyaluronan concentration estimation

Quantification of total extracellular hyaluronan, encompassing all molecular weight species secreted into the conditioned medium of hVFF, was performed using the Hyaluronan Quantikine ELISA Kit (R&D Systems, Cat# DHYAL0), in accordance with the manufacturer’s protocol. This assay is based on a quantitative sandwich enzyme-linked immunosorbent assay (ELISA) format, wherein recombinant human aggrecan (rhAggrecan) is pre-coated onto the wells of a microplate to serve as the capture reagent. Standards, controls, and conditioned medium samples were added to the wells, allowing hyaluronan present in the samples to bind to the immobilized rhAggrecan. After incubation, unbound components were removed through a series of washes, and an enzyme-linked rhAggrecan detection reagent was added to each well to form a sandwich complex with the captured hyaluronan. Following a second wash to eliminate excess detection reagent, a chromogenic substrate solution was introduced. The enzymatic reaction produced a colorimetric signal proportional to the concentration of hyaluronan in the sample, which was quantified by measuring absorbance using a microplate reader. To account for variations in cell number and metabolic activity, hyaluronan concentrations were normalized to total RNA content extracted from corresponding cell cultures. Data are expressed as mean ± standard deviation (SD) from three replicates.

### 2.8 Immunoblotting

Cell lysates were prepared and subjected to 10–12 % SDS-PAGE under reducing conditions to separate proteins by molecular weight. Proteins were then transferred onto nitrocellulose membranes using the iBlot™ Dry Blotting System (Thermo Fisher Scientific, USA) following the manufacturer’s instructions. Membranes were blocked for 1 h at room temperature in Tris-buffered saline (TBS) containing 0.1 % Tween-20 and 5 % bovine serum albumin (BSA) to minimize non-specific binding. After blocking, membranes were incubated overnight at 4 °C with primary antibodies diluted in TBS containing 0.1 % Tween-20, 1 % BSA, and 0.02 % sodium azide (NaN₃). The primary antibodies used were rabbit anti-HYAL2 (1:500; Cat# sc-99022, Santa Cruz Biotechnology, USA), rabbit anti-CD44 (1:1000; Cat# ab24504, Abcam, USA), and mouse anti-GAPDH (1:5000; Cat# sc-32233, Santa Cruz Biotechnology, USA). Following incubation, membranes were washed three times for 10 min each with TBS containing 0.1 % Tween-20 and then incubated for 1 h at room temperature with horseradish peroxidase (HRP)-conjugated secondary antibodies diluted in blocking buffer. Specifically, HRP-conjugated anti-rabbit IgG (1:3000; Cat# 7074, Cell Signaling Technology, USA) was used for HYAL2 and CD44, and HRP-conjugated goat anti-mouse IgG (1:5000; Cat# 91196, Cell Signaling Technology, USA) was used for GAPDH. After a final series of washes, protein bands were visualized using enhanced chemiluminescence (ECL) reagents (Thermo Fisher Scientific, USA) and imaged using a ChemiDoc™ Imaging System (Bio-Rad Laboratories, USA). Representative results are shown from two (n = 2) independent experimental replicates.

### 2.9 Transfection

CD44 mRNA expression in hVFF cells was transiently silenced via reverse transfection using gene-specific siRNA (Silencer™ Pre-Designed siRNA CD44; 20 nmol; Cat. No. AM16704, ID: 114068; ThermoFisher Scientific, USA) and a non-targeting control siRNA (Cat. No. sc-37007; Santa Cruz Biotechnology Inc., USA), each administered at a final concentration of 40 nM. Transfections were performed using Lipofectamine™ 3000 (Cat. No. L3000008; ThermoFisher Scientific, USA) per manufacturer’s protocol. Briefly, 3 × 10⁵ hVFF cells were suspended in complete growth medium lacking antibiotics, mixed with the transfection reagent, and seeded into 6-well tissue culture plates. After 48 h, the transfection medium was removed and substituted with complete medium with e-liquid (0.437 mg/mL) for 24 h.

### 2.10 Statistical analysis

The lethal concentration 50 (LC₅₀), defined as the concentration of a substance required to reduce cell viability by 50 %, was determined through concentration-response curve analysis and further validated using Probit regression analysis to ensure statistical robustness. Quantitative data from experimental assays were compiled and analyzed using Microsoft Excel and GraphPad Prism software (version 10.3.1). Graphs represent mean values ± standard deviation (SD) from three independent biological replicates unless otherwise specified. Statistical comparisons between treated and untreated groups were performed using a two-tailed paired Student’s t-test. Significance levels were denoted as follows: *p < 0.05, **p < 0.01, and ***p < 0.001, indicating increasing levels of statistical confidence in the observed differences.

## 3 Results

### 3.1 The effects of unvaporized e-liquid on hVFF cell morphology and viability

**Fig. 1:**
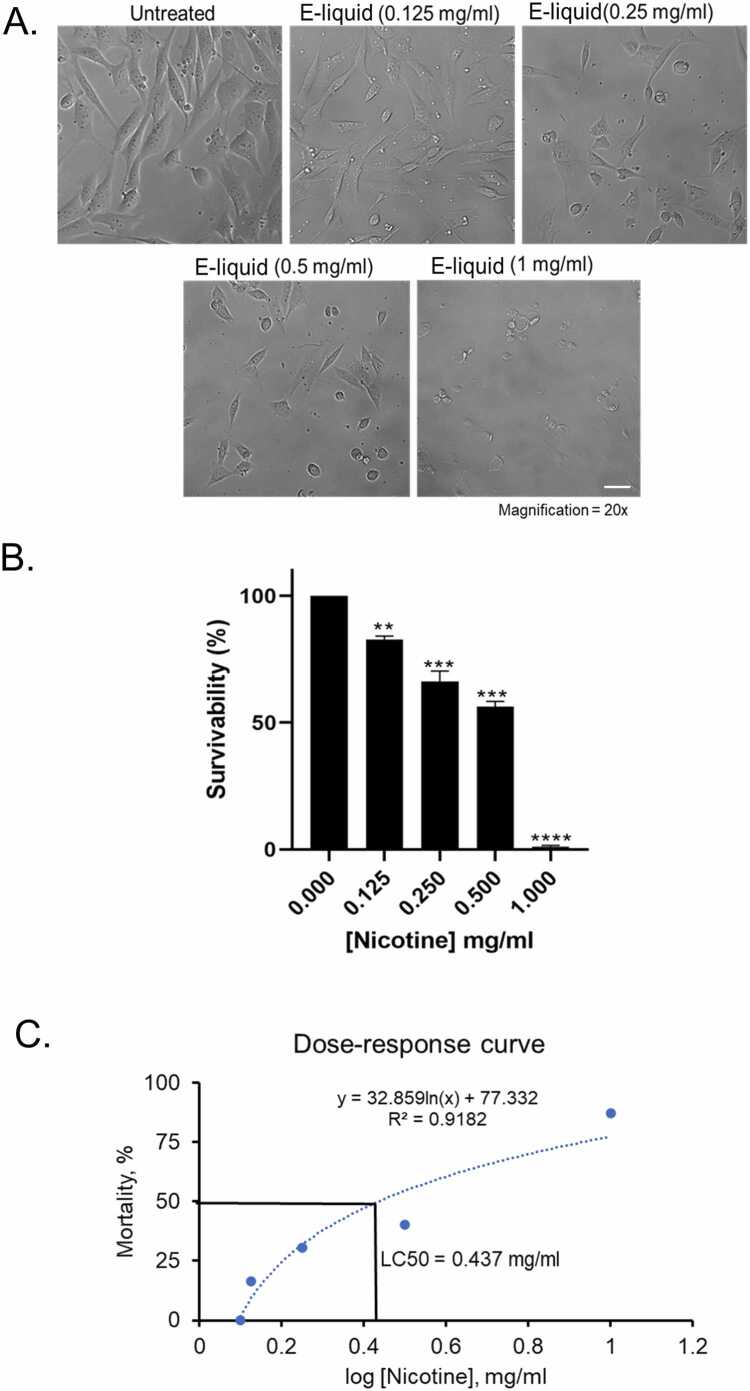
Effects of unvaporized e-liquid exposure on hVFF cell morphology and viability. (A) Representative brightfield micrographs (20 × magnification; scale bar = 100 µm) of hVFFs treated with e-liquid containing nicotine at concentrations of 0.125, 0.250, 0.5, and 1 mg/mL for 24 h (n = 4). (B) Cell viability was assessed using the WST-1 assay. Data are presented as mean ± standard deviation (SD) from three independent experiments (n = 3). Statistical significance was determined using unpaired two-tailed Student’s *t*-test: * p < 0.05, ** p < 0.01, *** p < 0.001, **** p < 0.0001. (C) Concentration–response curve showing the calculated LC₅₀ for e-liquid exposure. X-axis is plotted on a logarithmic scale.

The results of this study demonstrate a clear concentration-dependent cytotoxic effect of nicotine-containing e-liquid on human vocal fold fibroblasts (hVFF), as evidenced by both morphological changes and quantitative viability assays. Cells were treated with e-liquid at nicotine concentrations ranging from 0.125 mg/mL to 1 mg/mL, and brightfield microscopy revealed progressive structural damage correlating with increasing concentration (Fig. 1A). At 0.25 mg/mL, cells began to show signs of stress, while at 0.5 mg/mL, they adopted abnormal spindle-like and rounded morphologies, indicative of cytoskeletal disruption. At the highest concentration of 1 mg/mL, nearly all cells were nonviable, confirming the severe cytotoxicity of the e-liquid at this concentration. Quantitative analysis of cell survivability further supported these observations, showing a significant decline in viability across the concentration range (Fig. 1B). Specifically, cell viability decreased to approximately 75 % at 0.125 mg/mL (p < 0.01), 50 % at 0.25 mg/mL (p < 0.001), and 25 % at 0.5 mg/mL (p < 0.001), with complete cell death observed at 1 mg/mL (p < 0.0001). A dose-response curve was generated to model the relationship between nicotine concentration and cell mortality, with a high correlation coefficient (R² = 0.91), indicating strong predictive validity (Fig. 1C). The calculated LC_50_ value was 0.437 mg/mL, representing the concentration at which 50 % of the cell population was lethally affected. This value was further validated using Probit analysis and subsequently adopted as the optimal concentration for downstream experiments. These findings confirm the potent cytotoxicity of nicotine-containing e-liquid and establish a quantitative framework for evaluating its toxicological impact on vocal fold fibroblast viability.

### 3.2 Impact of e-liquid on intracellular ROS generation in hVFF

**Fig. 2:**
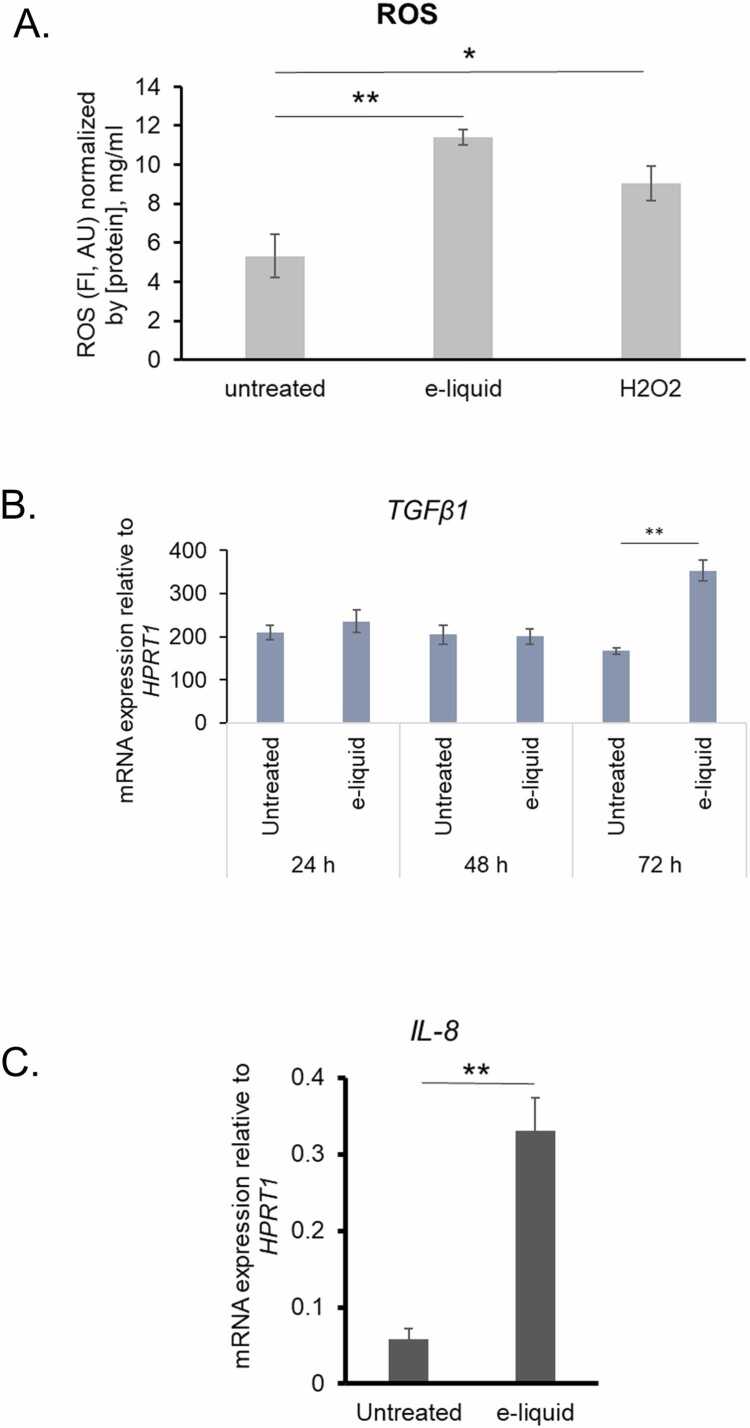
Effects of e-liquid exposure on reactive oxygen species (ROS) generation and inflammatory gene expression in hVFFs. (A) Intracellular ROS levels were measured following 72 h exposure to e-liquid (0.437 mg/mL). H₂O₂-treated cells served as a positive control. ROS levels are expressed as fluorescence intensity (FI, arbitrary units [AU]), normalized to total protein concentration (mg/mL). (B) Quantitative real-time PCR (qRT-PCR) analysis of TGF-β1 mRNA expression following 72 h e-liquid exposure. (C) qRT-PCR analysis of IL-8 mRNA expression following 72 h e-liquid exposure. Data are presented as mean ± standard deviation (SD) from three replicates (n = 3). Statistical significance was determined using unpaired two-tailed Student’s *t*-test: * p < 0.05, ** p < 0.01.

The results of this study reveal that exposure to nicotine-containing e-liquid induces oxidative stress and inflammatory responses in human vocal fold fibroblasts (hVFF), as evidenced by elevated reactive oxygen species (ROS) production and upregulated expression of key inflammatory genes. Quantitative analysis of ROS levels, normalized to protein concentration, showed a significant increase in the e-liquid treated group compared to untreated controls. Specifically, untreated cells exhibited ROS levels of approximately 5 arbitrary units (AU), while e-liquid exposure (0.437 mg/mL for 72 h) raised this to around 11 AU (p < 0.01). For comparison, cells treated with hydrogen peroxide (H₂O₂, 5 µM), a known oxidative stress inducer, showed ROS levels of approximately 8 AU, which were significantly lower than those induced by e-liquid (p < 0.05), suggesting that e-liquid may exert a stronger oxidative effect than H₂O₂ under these conditions (Fig. 2A).

### 3.3 Impact of e-liquid on mRNA expression of pro-inflammatory genes in hVFF

The mRNA expression of TGFβ1, a pro-fibrotic cytokine, was assessed at 24–72 h exposure to unvaporized e-liquid (0.437 mg/mL). There was no significant change in the expression level of TGFβ1 at 24–48 h (Fig. 2B). However, at 72 h, TGFβ1 expression in e-liquid treated cells (0.437 mg/mL) significantly increased compared to the untreated group (p < 0.01), indicating a time-dependent induction of fibrotic signaling (Fig. 2B). Increase in expression level of pro-inflammatory gene TGFβ1 prompted us to further validate it by detecting expression level of another pro-inflammatory chemokine, Interleukin 8 (IL-8). Because TGFβ1 elevated at 72 h, we measured the expression level of IL-8 only at one time point (i.e., 72 h). We observed that IL-8 was markedly upregulated in response to e-liquid exposure (0.437 mg/mL). Untreated cells showed baseline IL-8 expression, while e-liquid treated cells exhibited a more than threefold increase (p < 0.01), highlighting a robust inflammatory response (Fig. 2C).

### 3.4 Effects of e-liquid on collagen and collagen-regulating gene expression in hVFF

**Fig. 3:**
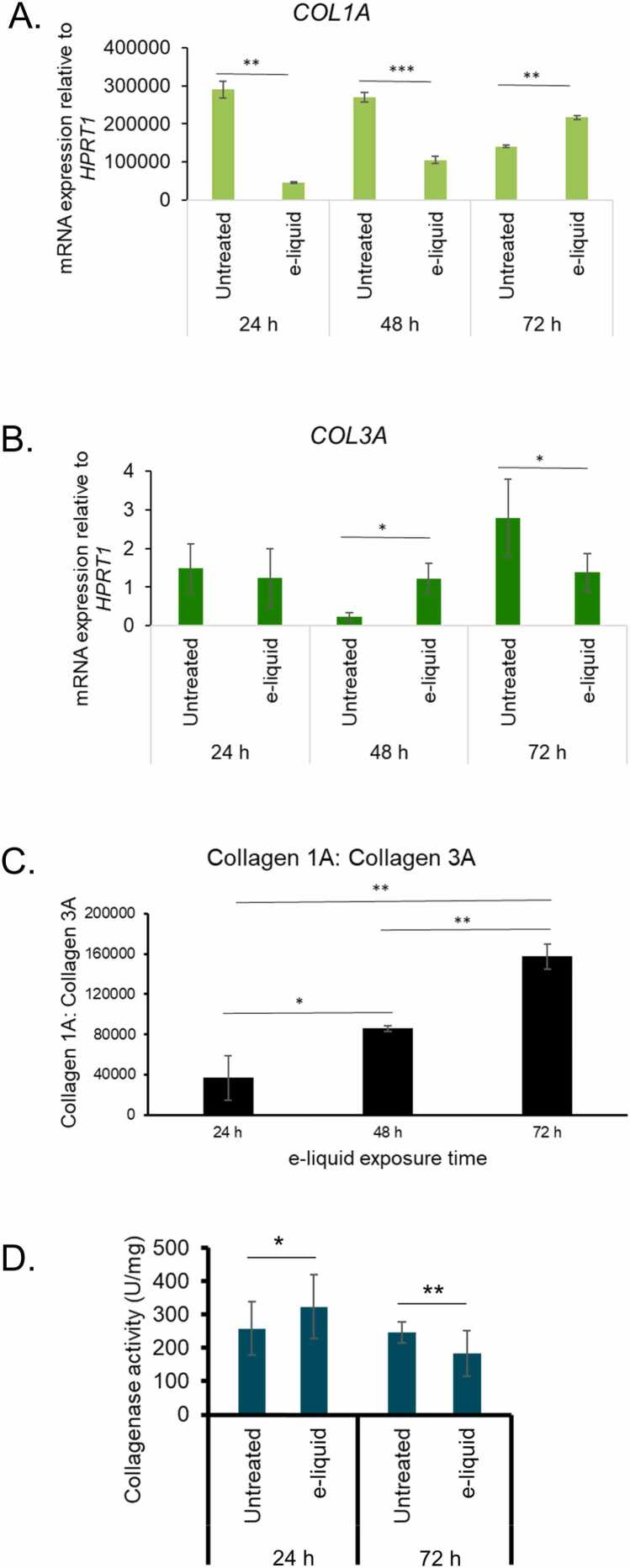
E-liquid exposure alters collagen-regulating gene expression and collagenase activity in hVFFs. (A, B) mRNA expression levels of collagen type I alpha 1 (*COL1A1*) and collagen type III alpha 1 (*COL3A1*) were quantified by qRT-PCR following e-liquid exposure (0.437 mg/mL) for 24, 48, and 72 h. (C) The *COL1A1*:*COL3A1* expression ratio was calculated at each time point to assess collagen remodeling dynamics. (D) Collagenase activity was measured using a collagenase assay kit after short-term (24 h) and long-term (72 h) e-liquid exposure. Data are presented as mean ± standard deviation (SD) from three replicates (n = 3). Statistical significance was determined using unpaired two-tailed Student’s *t*-test: * p < 0.05, ** p < 0.01.

The results of this study demonstrate a dynamic and time-dependent modulation of collagen-related gene expression and enzymatic activity in human vocal fold fibroblasts (hVFF) following e-liquid exposure (0.437 mg/mL). Initial analysis revealed that *COL1A1* mRNA, encoding collagen type I alpha, was expressed at significantly higher levels than *COL3A1*, which encodes collagen type III alpha, under baseline conditions. Upon 24 h exposure to e-liquid, *COL1A1* expression was significantly reduced (p < 0.01), indicating an acute suppressive effect on collagen type I synthesis, while *COL3A1* levels remained unchanged, suggesting selective transcriptional sensitivity (Figs. 3A, 3B). Concurrently, collagenase activity, responsible for collagen degradation, was elevated (p < 0.05), pointing to an active collagenolytic response during early exposure (Fig. 3D). However, with prolonged exposure, a reversal in this pattern was observed: collagenase activity was suppressed, and *COL1A1* expression increased, while *COL3A1* expression declined (Figs. 3A, 3B, 3D). This shift implies a disruption in collagen turnover, favoring accumulation of collagen type I and reduced degradation capacity. The ratio of *COL1A1* to *COL3A1* rose significantly over time (Fig. 3C), reinforcing the notion that chronic e-liquid exposure skews the collagen profile toward a fibrotic phenotype. These findings suggest that while short-term exposure activates collagen degradation pathways, long-term exposure promotes collagen deposition and may contribute to extracellular matrix remodeling in hVFF cells.

### 3.5 Effect of unvaporized e-liquid exposure on total extracellular hyaluronan level

**Fig. 4:**
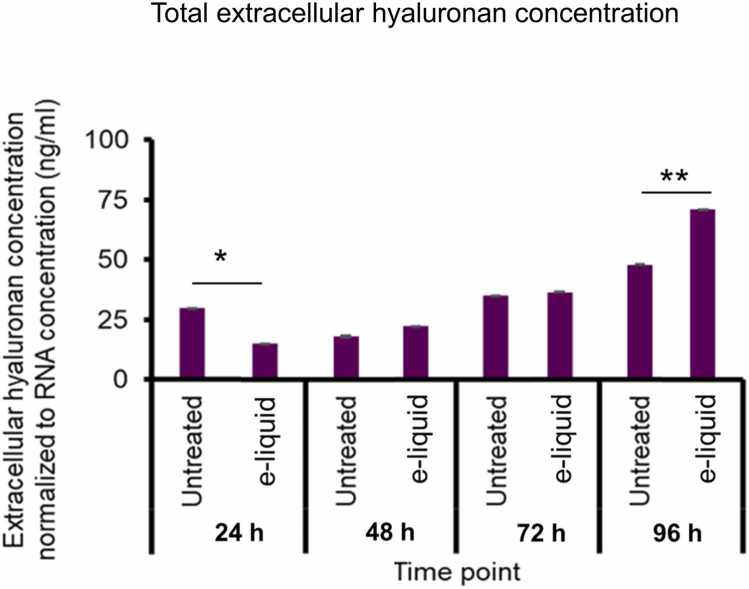
E-liquid exposure modulates extracellular hyaluronan release in hVFFs. Total extracellular hyaluronan concentration in conditioned medium was measured at 24, 48, 72, and 96 h following e-liquid exposure (0.437 mg/mL) using a hyaluronan-specific ELISA. Hyaluronan levels were normalized to total RNA concentration. Data are presented as mean ± standard deviation (SD) from three replicates (n = 3). Statistical significance was determined using unpaired two-tailed Student’s *t*-test: * p < 0.05, ** p < 0.01.

To assess the impact of e-liquid exposure (0.437 mg/mL) on extracellular hyaluronan production, we quantified hyaluronan concentrations in the culture medium at 24, 48, 72, and 96 h, normalizing the values to total RNA content to account for cell density and viability. At 24 h, e-liquid-treated cells exhibited a significant reduction in extracellular hyaluronan levels, measuring 10.3 ± 1.2 ng/mL compared to 25.1 ± 2.0 ng/mL in untreated controls (*p* < 0.05) (Fig. 4). This early suppression suggests an acute inhibitory effect of e-liquid constituents on hyaluronan synthesis or secretion. By 48 h, the trend reversed, with e-liquid-treated cells producing slightly more hyaluronan (24.8 ± 2.3 ng/mL) than untreated cells (20.2 ± 1.9 ng/mL), although this difference was not statistically significant. At 72 h, hyaluronan levels continued to rise in both groups, reaching 35.1 ± 2.7 ng/mL in treated cells and 30.3 ± 2.5 ng/mL in controls. At 96 h, a pronounced increase was observed in the e-liquid-exposed group, which reached 75.2 ± 4.1 ng/mL compared to 50.4 ± 3.6 ng/mL in untreated cells (p < 0.01) (Fig. 4). Notably, untreated hVFF also showed a steady increase in total extracellular hyaluronan from 27.0 ng/mL at baseline to 50.4 ng/mL at 96 h, likely reflecting cell proliferation and matrix accumulation under normal growth conditions. This baseline trend underscores the dynamic nature of hyaluronan turnover in vocal fold fibroblasts and provides a reference for interpreting the biphasic response observed in e-liquid-treated cells.

These findings demonstrate that e-liquid exposure modulates extracellular hyaluronan levels in a time-dependent manner—initial suppression followed by progressive induction. The significant increase at 96 h may reflect enhanced activity of hyaluronan synthase enzymes or altered turnover dynamics, potentially contributing to extracellular matrix remodeling and inflammatory signaling in exposed tissues.

### 3.6 Effects of e-liquid exposure on transcription of hyaluronan synthase genes

**Fig. 5:**
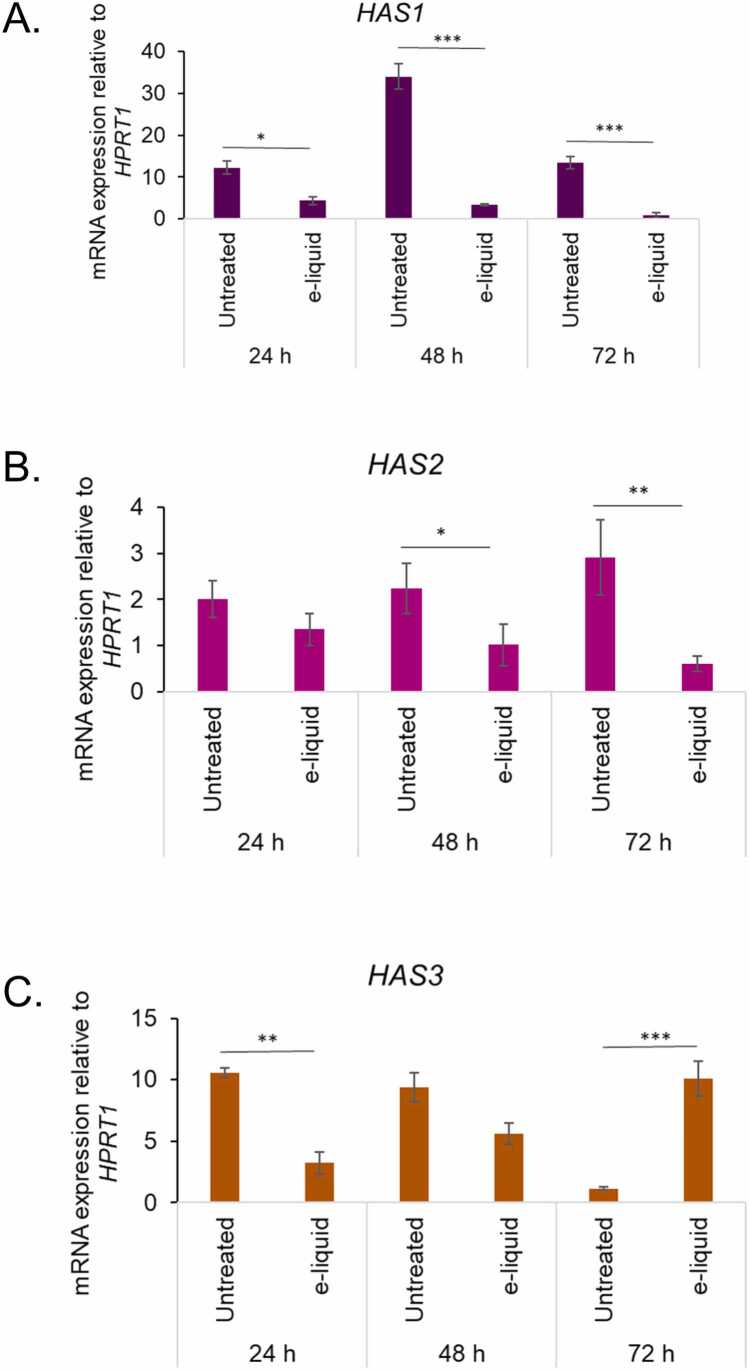
E-liquid exposure alters mRNA expression of hyaluronan synthases in hVFFs. Quantitative real-time PCR (qRT-PCR) was used to assess mRNA expression levels of hyaluronan synthase isoforms *HAS1* (A), *HAS2* (B), and *HAS3* (C) following e-liquid exposure (0.437 mg/mL) for 24 h (short-term), 48 h, and 72 h (long-term). Data are presented as mean ± standard deviation (SD) from three replicates (n = 3). Statistical significance was determined using unpaired two-tailed Student’s *t*-test: * p < 0.05, ** p < 0.01, *** p < 0.001.

We investigated the effects of e-liquid exposure on hyaluronan biosynthesis by analyzing the expression of the hyaluronan synthase genes *HAS1*, *HAS2*, and *HAS3* using quantitative real-time PCR (qRT-PCR). Cells were exposed to e-liquid (0.437 mg/mL) for 24, 48, and 72 h, and compared to untreated controls. Our analysis revealed a consistent and significant downregulation of *HAS1* across all time points. At 24 h, *HAS1* expression decreased from 10.2 ± 1.1 units in untreated cells to 2.6 ± 0.4 units in treated cells (*p* < 0.05). This suppression intensified at 48 h, with untreated cells expressing 35.4 ± 2.8 units compared to only 2.4 ± 0.3 units in exposed cells (*p* < 0.001). By 72 h, *HAS1* levels dropped further to 1.0 ± 0.2 units in treated cells versus 10.1 ± 0.9 units in controls (*p* < 0.001), indicating sustained transcriptional repression (Fig. 5A).

Expression of *HAS2* was moderately affected by e-liquid exposure. At 24 h, levels declined, though this change was not statistically significant. However, at 48 h, *HAS2* expression was significantly reduced from 2.0 ± 0.3 units in untreated cells to 1.0 ± 0.2 units in treated cells (*p* < 0.05). A more pronounced suppression was observed at 72 h, with expression falling from 3.1 ± 0.4 units to 1.5 ± 0.3 units (*p* < 0.01) (Fig. 5B). In contrast, *HAS3* exhibited a biphasic response to e-liquid exposure. At 24 h, expression was significantly reduced from 11.2 ± 1.0 units in control cells to 4.1 ± 0.6 units in treated cells (*p* < 0.01). A similar trend was observed at 48 h, with a decrease from 8.3 ± 0.9 units to 4.2 ± 0.5 units, although this difference did not reach statistical significance. Notably, at 72 h, *HAS3* expression was significantly upregulated almost 7-fold in e-liquid-treated cells, compared to untreated controls (*p* < 0.001) (Fig. 5C). This late-stage induction suggests a compensatory mechanism or stress-adaptive response specific to *HAS3*.

Overall, these findings demonstrate that e-liquid exposure disrupts hyaluronan synthase gene expression in a time-dependent and gene-specific manner. The persistent downregulation of *HAS1* and *HAS2* may impair HMW HA production, while the delayed upregulation of *HAS3* could reflect increase in LMW HA production under prolonged chemical stress.

### 3.7 Effect of e-liquid on CD44-dependent hyaluronan catabolic gene expression

**Fig. 6:**
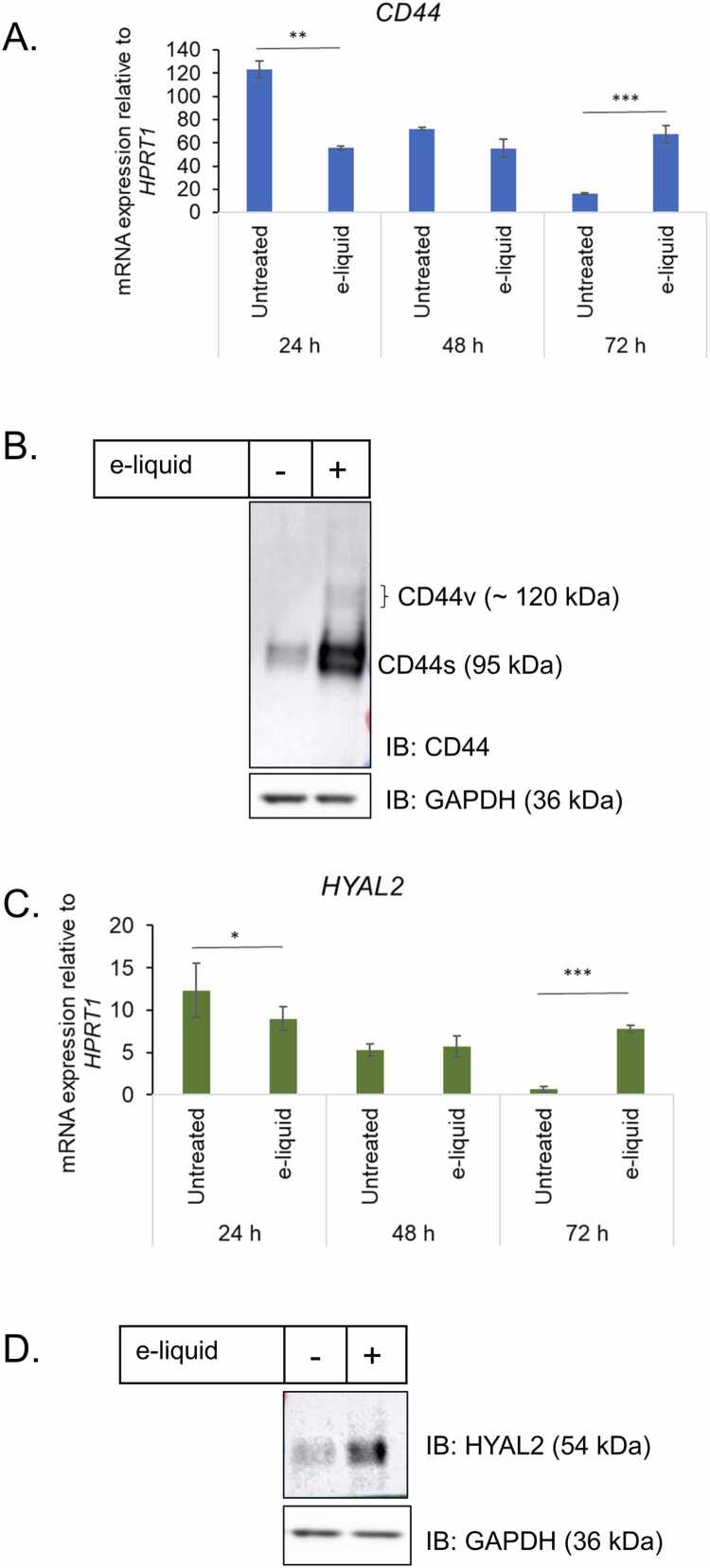
E-liquid exposure modulates CD44 and HYAL2 expression in hVFFs. (A, C) mRNA expression levels of *CD44* and *HYAL2* were quantified by qRT-PCR following e-liquid exposure (0.437 mg/mL). (B, D) Corresponding protein levels were assessed by immunoblotting. qRT-PCR data are presented as mean ± standard deviation (SD) from three independent experiments (n = 3). Statistical significance was determined using unpaired two-tailed Student’s *t*-test: * p < 0.05, ** p < 0.01, *** p < 0.001. Immunoblots shown are representative of two biological replicates.

We examined the impact of e-liquid exposure (0.437 mg/mL) on genes involved in the CD44-dependent hyaluronan catabolism pathway, specifically *CD44* and *HYAL2*. qRT-PCR analysis revealed that a 24-h exposure to e-liquid significantly suppressed *CD44* and *HYAL2* mRNA levels by approximately 2.3-fold (p < 0.01) and 1.8-fold (p < 0.05), respectively, compared to untreated controls. Their expression returned to baseline after 48 h, showing no significant difference from untreated cells. In contrast, a 72-h exposure resulted in a marked increase in mRNA levels: *CD44* was upregulated by 2.7-fold and *HYAL2* by 2.4-fold (both p < 0.001) (Fig. 6A & 6 C).

Immunoblotting further confirmed that long-term e-liquid exposure (0.437 mg/mL) significantly elevated the protein expression of both genes (Fig. 6B & 6D). Densitometric analysis normalized to GAPDH showed a 2.5-fold increase in CD44 protein and a 2.1-fold increase in HYAL2 protein levels at 72 h (p < 0.01 for both). We observed increased expression of CD44s isoforms (∼95 kDa) under e-liquid exposure (0.437 mg/mL). Additionally, CD44v isoforms (∼120 kDa), which were not detectable under untreated conditions, were clearly expressed in e-liquid-treated cells (Fig. 6B), suggesting a shift in isoform regulation potentially linked to cellular stress or phenotypic adaptation.

### 3.8 Impact of CD44 on IL-8 mRNA expression in e-liquid-treated hVFF

**Fig. 7:**
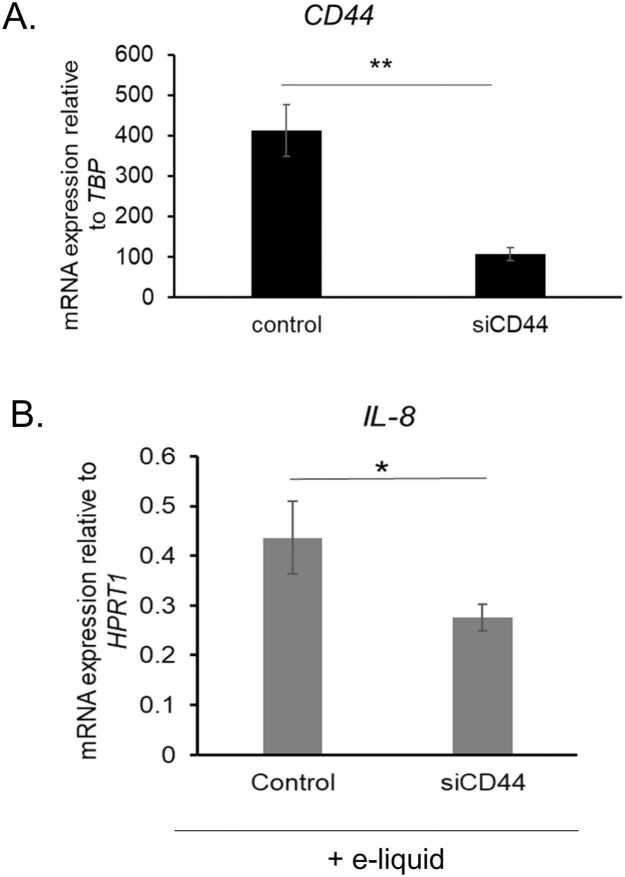
CD44 silencing downregulates *IL-8* mRNA expression in hVFFs exposed to unvaporized e-liquid. (A) CD44 mRNA levels were quantified by qRT-PCR following siRNA-mediated knockdown. CD44 expression was significantly reduced (∼4-fold) compared to scrambled siRNA controls (p < 0.01). (B) hVFFs with intact or silenced CD44 were treated with unvaporized e-liquid containing 0.437 mg/mL nicotine for 24 h. IL-8 mRNA expression was measured by qRT-PCR. CD44 knockdown significantly decreased IL-8 expression relative to control cells (p < 0.05). Data are presented as mean ± SD from three replicates. Statistical significance was determined using unpaired two-tailed Student’s *t*-test: * p < 0.05, ** p < 0.01

Upregulation of CD44 and HYAL2 suggests that hyaluronan might be degraded via a CD44-dependent catabolic pathway in hVFFs exposed to unvaporized e-liquid (0.437 mg/mL). To examine CD44’s role in inflammation, *IL-8* mRNA expression was quantified in hVFFs with intact CD44 expression and in cells with CD44 silenced via siRNA, following 24 h of e-liquid exposure. CD44 knockdown significantly reduced IL-8 transcript levels compared to controls (p < 0.05) (Fig. 7), underscoring CD44’s involvement in hyaluronan-mediated inflammatory signaling in e-liquid–treated hVFFs.

## 4 Discussion

This study presents the first comprehensive analysis of how e-liquid exposure modulates extracellular hyaluronan regulation in human vocal fold fibroblasts (hVFFs), revealing time-dependent shifts in hyaluronan metabolic gene expression. Given hyaluronan’s central role in vocal fold biomechanics, contributing to viscosity, pliability, and phonatory efficiency, these findings offer critical insight into the potential toxicological impact of e-cigarette constituents on vocal fold health. Short-term exposure (24 h) to unvaporized e-liquid resulted in a significant reduction in extracellular hyaluronan concentration (from 27 ng/mL to 15 ng/mL), accompanied by downregulation of hyaluronan synthesizing genes HAS1, HAS2, and HAS3. In contrast, prolonged exposure (72–96 h) induced a distinct metabolic shift: HAS1 and HAS2, which primarily synthesize anti-inflammatory high molecular weight hyaluronan (HMW-HA), remained suppressed, while HAS3—associated with pro-inflammatory low molecular weight hyaluronan (LMW-HA) synthesis,was upregulated. This was accompanied by increased expression of catabolic genes HYAL2 and CD44, resulting in elevated total hyaluronan levels (from 50 ng/mL to 75 ng/mL). These findings suggest that enhanced HAS3 and hyaluronan catabolic gene expression together promote LMW-HA accumulation, a known driver of inflammation and tissue remodeling. Finally, silencing CD44 in hVFFs significantly downregulates pro-inflammatory IL-8 transcription following exposure to unvaporized e-liquid, underscores importance of CD44-dependent hyaluronan catabolism and signaling in regulating vocal fold inflammation. The essence of these observations is that these mechanisms, particularly the ROS-induced increase in HYAL2 and its association with CD44, can cause a shift in the ECM composition from predominantly high-molecular weight hyaluronan content towards LMW HA. Thus, targeting the mechanisms of LMW HA enrichment in the vocal fold submucosa could contribute to a therapeutic strategy.

**Fig. 8:**
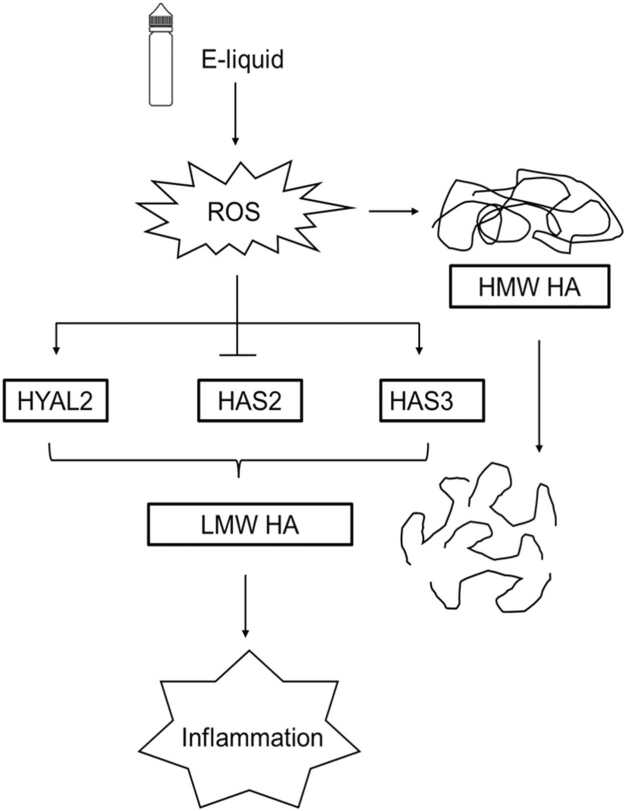
Proposed mechanistic model illustrating the role of hyaluronan signaling in hVFFs following long-term unvaporized e-liquid exposure. Prolonged exposure to unvaporized e-liquid (0.437 mg/mL) induces intracellular reactive oxygen species (ROS), contributing to the generation of low molecular weight hyaluronan (LMW-HA). LMW-HA may arise from oxidative degradation of high molecular weight hyaluronan (HMW-HA), reduced expression of HAS2, and elevated expression of HAS3 and HYAL2. Accumulation of LMW-HA promotes a proinflammatory response in hVFFs, contributing to tissue remodeling and inflammatory activation.

Fig. 8 presents a mechanistic illustration of how exposure to e-liquid initiates a cascade of biochemical events contributing to inflammation in the human laryngeal epithelial submucosa, specifically through the generation of reactive oxygen species (ROS) and the enzymatic degradation of hyaluronan. Upon contact with cellular systems, e-liquid induces the production of ROS, which serves as a key mediator of oxidative stress. These ROS interact with HMW HA, a critical component of the extracellular matrix known for its anti-inflammatory and structural properties. The interaction results in the fragmentation of HMW HA into LMW HA. The enzymatic conversion is facilitated by three key enzymes: hyaluronidase 2 (HYAL2), hyaluronan synthase 2 (HAS2), and hyaluronan synthase 3 (HAS3). These enzymes are upregulated in response to ROS and contribute to the breakdown of HMW HA. The accumulation of LMW HA is associated with pro-inflammatory signaling, culminating in the activation of inflammatory pathways. This visual model underscores the toxicological relevance of e-liquid exposure, linking oxidative stress to extracellular matrix remodeling and inflammation. It provides a clear molecular framework for understanding how vaping-related compounds may compromise tissue integrity and promote inflammatory responses, which are critical considerations in the context of airway toxicity and disease progression.

The biphasic hyaluronan response underscores the complexity of ECM regulation under toxic stress. While initial exposure suppresses hyaluronan synthesis, extended exposure appears to trigger a pro-inflammatory remodeling phase, potentially mediated by reactive oxygen species (ROS) and altered transcriptional control. Hyaluronan is the most abundant glycosaminoglycan in the vocal fold lamina propria, contributing to its viscoelastic properties and facilitating efficient phonation. Although e-cigarettes usage has surged globally, research on their toxic effects on vocal folds is limited [38]. E-cigarette exposure causes hyperplasia and metaplasia in the laryngeal mucosa [39]. It damages epithelium of the vocal fold mucosa, disrupting homeostasis and barrier function and prompting significant mucosal remodeling [40]. Long-term vapor exposure, unlike short-term exposure, induced inflammation in *in-vivo* studies, supporting our findings and highlighting the significance of vapor exposure duration for vocal fold damage [41], [42].

Importantly, we opted to use unvaporized e-liquid rather than vaporized forms, which are known to generate additional toxicants such as formaldehyde, acetaldehyde, and acrolein during thermal degradation [22], [23]. While this choice limits direct comparison to inhalation models, it isolates the effects of e-liquid constituents and avoids confounding by combustion byproducts. The use of unvaporized e-liquid in this study, while methodologically advantageous for controlled dosing and mechanistic insight, presents inherent limitations in terms of translational relevance. Vaporization alters the chemical profile of e-liquids through thermal degradation, potentially generating reactive carbonyls, free radicals, and metal particles from device components. These aerosolized byproducts are absent in unvaporized formulations and may contribute significantly to the toxicological burden during actual inhalation. Additionally, direct application of e-liquid to cell cultures may introduce non-physiological conditions, such as altered pH or osmolality, which could confound cellular responses. While our findings provide foundational data on ingredient-level toxicity, future studies incorporating aerosol generation and physiologically relevant exposure models are warranted to fully assess the health risks associated with e-cigarette use.

To date, only one study investigated e-cigarette effects on hVFF, but did not explore hyaluronan-regulating gene expression [43]. This study showed no significant changes in the expression of ECM and inflammatory genes after 24 h of exposure despite using more toxic vaporized e-liquids at higher nicotine concentrations (50, 25, and 12.5 mg/mL) [43]. Conversely, by applying less toxic, unvaporized e-liquids at lower concentrations (0.437 mg/mL nicotine), we also did not see inflammation but observed a significant change in ECM gene expression, in particular, genes regulating hyaluronan and collagen. Notably, both studies used the hVFF cell line from the same source [43]. We speculate that the vaporization of e-liquid might produce unknown components that affected ECM gene expression [43]. Different brands of e-liquids or e-cigarettes might also have varying effects on hVFF. We cannot rule out the possibility of using different clones of hVFF cells, which could also lead to differing responses.

Seventy-two hour- e-liquid exposure also induced collagen remodeling, with increased *COL1A1* expression and reduced collagenolysis, leading to ECM stiffening. The rising *COL1A1*: *COL3A1* ratio suggests a shift toward more rigid fibrillar collagen, which is associated with fibrotic and inflammatory phenotypes. Concurrent upregulation of CD44s and CD44v isoforms further implicates CD44-mediated signaling in ECM remodeling. While CD44 binds hyaluronan and collagen, its role in inflammation appears context dependent. Notably, CD44 deletion in cutaneous wound models increased inflammation and collagen accumulation [31], [32], [33], contrasting with our findings. This may be attributed to the structural and functional differences between skin and laryngeal mucosa. Skin possesses a cornified, stratified epithelium, whereas the larynx is lined by a predominantly pseudostratified respiratory epithelium, which may differ in key mechanisms of toxicity and inflammation. Our finding suggests that e-liquid toxicity may activate alternative CD44v-dependent pathways in hVFF.

This study demonstrates that CD44 silencing in human vocal fold fibroblasts (hVFFs) significantly downregulates *IL-8* mRNA expression following exposure to unvaporized nicotine-containing e-liquid. IL-8 (CXCL8) is a proinflammatory chemokine regulated by ECM dynamics and cellular stress. The reduction in IL-8 suggests that CD44 may act as a positive regulator of inflammation in response to xenobiotic exposure. As the principal receptor for hyaluronan, CD44 facilitates hyaluronan internalization and degradation via hyaluronidases such as Hyal-1 and Hyal-2. Its silencing disrupts hyaluronan catabolism, leading to retention of HMW-HA, which exerts anti-inflammatory effects by suppressing cytokine expression and NF-κB signaling [44], [45]. This shift may dampen IL-8–mediated inflammatory responses. Supporting evidence indicates that CD44 loss reduces Src and JAK kinase activity [46], [47], and that HMW-HA attenuates matrix metalloproteinase expression via CD44-dependent pathways [45], suggesting a broader role for CD44 in modulating fibroblast reactivity to aerosolized toxicants. Collectively, these findings suggest that CD44 is a critical mediator of fibroblast sensitivity to environmental stressors and that its loss may suppress chemokine-driven inflammation by altering hyaluronan dynamics. Targeting CD44–hyaluronan interactions may offer therapeutic potential for mitigating aerosol-induced inflammatory responses in the vocal fold microenvironment.

This pilot study represents Phase I of a novel drug discovery initiative targeting vocal fold inflammation through modulation of hyaluronan metabolism. Using immortalized human vocal fold fibroblasts (hVFFs), we investigated the cellular response to unvaporized e-liquid exposure, establishing a foundational model for hyaluronan-mediated inflammatory signaling. Insights from this phase will inform the design of Phase II, which will expand the scope by evaluating vaporized e-liquids from diverse flavors and commercial brands in primary hVFFs, thereby enhancing translational relevance and therapeutic potential. It is also noteworthy that hVFFs in the lamina propria are indirectly exposed to the constituents that are mainly passing through the epithelial cells or follow a paracellular transport through intercellular spaces if the epithelial permeability is increased. In consequence, the hVFFs are not directly exposed to the e-liquid or its aerosol droplets. In addition, the inflammatory mediators generated by the exposed epithelial cells and the activated immune cells present in the larynx mucosa *in vivo* may further enhance or modulate the hyaluronan and collagen turnover in the lamina propria. Hence, further exploring their contributions to the mechanism of action would be an important next step, using either combinatory 3-dimensional *in vitro* models, or a rodent *in vivo* model, and leveraging the identified genes of the hyaluronan synthesis and degradation as biomarkers, corroborated by actual measurement of the resulting HMW-LMW hyaluronan distribution.

## 5 Limitations

Vibrational stimuli significantly influence CD44 expression in hVFF [48]. Future studies incorporating dynamic mechanical stimulation would better replicate the in vivo vocal fold environment and clarify the interplay between biomechanical forces and ECM remodeling under toxic stress. Secondly, we did not assess hyaluronan molecular weight distribution, which is critical for distinguishing between HMW and LMW HA-mediated effects. Third, the hVFF cell line used was immortalized via hTERT transduction, which may alter endogenous p53 activity—a known regulator of HAS2 and HAS3 transcription [28], [49]. Delayed hyaluronan accumulation observed at 96 h may reflect p53-mediated suppression of hyaluronan synthesis, necessitating validation in primary hVFF cells.

## 6 Conclusion

In summary, this study is the first to examine the toxic effects of e-liquids on metabolic genes regulating hyaluronan levels in hVFF. These findings suggest a mechanistic link between e-liquid-induced inflammation and altered extracellular matrix remodeling in hVFF. Preliminary findings indicate that prolonged e-liquid exposure leads to inflammation and elevates the expression of genes regulating hyaluronan catabolism and LMW HA production in hVFF, suggesting a plausible link between these two cellular events. Thus, targeting hyaluronan degradation and LMW HA production could be a promising therapeutic strategy to alleviate vocal fold scarring by e-cigarette toxicity.

## CRediT authorship contribution statement

**Kaustuv Basu:** Writing – review & editing, Writing – original draft, Validation, Supervision, Project administration, Methodology, Investigation, Formal analysis, Data curation, Conceptualization. **James Li:** Writing – review & editing, Investigation. **Luc Mongeau:** Writing – review & editing, Resources, Project administration, Funding acquisition.

## Declaration of Competing Interest

The authors declare that they have no known competing financial interests or personal relationships that could have appeared to influence the work reported in this paper.

## Data Availability

The data that has been used is confidential.
